# Measuring aortic distensibility with cmr using central pressures estimated in the magnet: comparison with carotid and peripheral pressures

**DOI:** 10.1186/1532-429X-13-S1-P27

**Published:** 2011-02-02

**Authors:** Alban Redheuil, Mourad Bensalah, Nadjia Kachenoura, Eric Bruguiere, Arshid Azarine, Ludivine Perdrix, Erwan Bozec, Pierre Boutouyrie, Alain DeCesare, Elie Mousseaux

**Affiliations:** 1 University of Paris Descartes-European Hospital Georges Pompidou and INSERM U678, Paris, France

## Objective

To evaluate the feasibility and consequences on local aortic distensibility estimation of using central pressure measurement in the magnet, simultaneous to aortic imaging with CMR

## Background

Several studies have demonstrated the feasibility and value of studying local aortic strain with CMR. Calculating aortic distensibility ideally requires the knowledge of simultaneously acquired central pressure changes which until recently remained a challenge during CMR. New MR-compatible devices using an oscillometric technique to estimate central pressures from a brachial cuff are now available but poorly evaluated in this setting.

## Methods

We studied 49 subjects (26 men, 23 women, age 44±18 years) free from overt cardiovascular disease. Ascending aortic strain was determined by CMR using an automated segmentation of SSFP cine acquisitions. Central pressures were estimated from 1) carotid pressures measured immediately after the CMR exam using applanation tonometry and 2) brachial cuff pressure measured simultaneously with aortic cine imaging in the magnet, using the Vicorder™ Device . In both cases, mean brachial pressures was integrated in the calculation of central pressures after applying the transfert function. Central pressures were used to calculate the aortic distensibility defined as the ratio between aortic strain and central pulse pressure (AAD-carotid for carotid pressure and AAD-vicorder for the Vicorder device pressure) and applanation tonometry was further used to estimate the carotid augmentation index (AIx) and Carotid-femoral pulse wave velocity (cfPWV).

## Results

Average±SD systolic brachial, carotid and Vicorder pressures were respectively: 114±13, 105±13, 106±14mmHg. We found a strong linear relationship between AAD-carotid and AAD-vicorder (β=0.89, R2=0.91, p<0.001) with however a larger spread between values at higher pressures. The mean distensibility difference between the two methods was: -1.1±12 mmHg-1 and variability 0.9%. Expectedly, distensibility values measured using peripheral brachial cuff pressures were lower than using either central pressures due to the amplification phenomenon in relation to vascular aging (Table).

**Table 1:** Average ascending aortic distensibilities according to central pressure measurement technique and age group

Ascending Aortic Distensibilities, mmHg-1.10-2	Age<50 years n=26	Age>50 years n=23
AAD peripheral(Brachial)	65±29	24±13
AAD central Carotid	80±34	31±17
AAD central Vicorder	83±37	30±18

The correlations between local aortic distensibility with age, AIx and cfPWV were significantly higher when using AAD-vicorder (respectively: r=-0.82, r=-0.62; r=0.61; p<0.001) than when using AAD-carotid (r=-0.79, r=-0.50, r=-0.58; p<0.001).

## Conclusions

Aortic distensibility may be measured by CMR using central pressures measured directly in the magnet, simultaneously with cine acquisitions. Resulting distensibilities are closely related to those using carotid pressures measured by tonometry outside the magnet and achieve higher correlation with age and markers of global aortic stiffness such as AIx and cfPWV.

